# Stomata at the crossroad of molecular interaction between biotic and abiotic stress responses in plants

**DOI:** 10.3389/fpls.2022.1031891

**Published:** 2022-10-14

**Authors:** Pengshuai Peng, Rui Li, Zhong-Hua Chen, Yuanyuan Wang

**Affiliations:** ^1^ Hubei Insect Resources Utilization and Sustainable Pest Management Key Laboratory, College of Plant Science and Technology, Huazhong Agricultural University, Wuhan, China; ^2^ School of Science, Western Sydney University, Penrith, NSW, Australia; ^3^ Hawkesbury Institute for the Environment, Western Sydney University, Penrith, NSW, Australia

**Keywords:** guard cell signaling, jasmonic acid, salicylic acid, abscisic acid, insect herbivory, plant innate immunity, abiotic stress

## Abstract

Increasing global food production is threatened by harsh environmental conditions along with biotic stresses, requiring massive new research into integrated stress resistance in plants. Stomata play a pivotal role in response to many biotic and abiotic stresses, but their orchestrated interactions at the molecular, physiological, and biochemical levels were less investigated. Here, we reviewed the influence of drought, pathogen, and insect herbivory on stomata to provide a comprehensive overview in the context of stomatal regulation. We also summarized the molecular mechanisms of stomatal response triggered by these stresses. To further investigate the effect of stomata–herbivore interaction at a transcriptional level, integrated transcriptome studies from different plant species attacked by different pests revealed evidence of the crosstalk between abiotic and biotic stress. Comprehensive understanding of the involvement of stomata in some plant–herbivore interactions may be an essential step towards herbivores’ manipulation of plants, which provides insights for the development of integrated pest management strategies. Moreover, we proposed that stomata can function as important modulators of plant response to stress combination, representing an exciting frontier of plant science with a broad and precise view of plant biotic interactions.

## Introduction

Climate change and food security are major global issues in the 21st century. The United Nations Sustainable Development Goals (SDGs) aimed to achieve 17 individual goals by 2030. SDG 2, ‘Zero Hunger’, is hinged on food security and improved nutrition. However, more than 800 million people were reported to have no sufficient food in 2021 across the world (FAO et al., 2022). More frequent climate extremes disrupt agricultural production, leading to persistent threats of starvation (Hatfield and Prueger, 2015). Increasing crop yield demands under harsh environmental conditions require massive upgraded new research efforts for plant stress resistance.

In the natural environment, plants often suffer from numerous abiotic stresses such as drought (Wang X. et al., 2018), waterlogging (Wang et al., 2017), heat (Sadok et al., 2021), chilling (Penfield et al., 2021), light (Cai et al., 2021), salinity (Tyerman et al., 2019), heavy metal (Hu et al., 2020), and metalloid stresses (Peleg and Blumwald, 2011; Deng et al., 2021). Meanwhile, climate change-induced outbreaks of insects and pathogens expose crops to unpredictable biotic stresses (Groll et al., 2008; Deutsch et al., 2018; Havko et al., 2020; Ristaino et al., 2021; Lin et al., 2022). Moreover, combinations of abiotic and biotic stresses may cause a trade-off for plant adaptability (Pandey et al., 2015). Therefore, understanding how plants respond to variable environments inundated with stresses is vital for improving crop productivity and quality.

Stomatal movement in the leaf epidermis is controlled by guard cells in most plants and guard cells and subsidiary cells in monocots, allowing terrestrial plants to balance between photosynthetic CO_2_ uptake and water loss (Hetherington & Woodward, 2003). Many studies have revealed the pivotal role of stomata in orchestrating interactions between biotic and abiotic stresses (Hetherington and Woodward, 2003; Berry et al., 2010; Nunes et al., 2020). The emission of CO_2_ is the main factor for the warmer average global temperatures, which could also induce the imbalance between photosynthesis and stomatal response affecting the water use efficiency of plants (Meinshausen et al., 2009). To provide a comprehensive overview of stomatal response to abiotic and biotic stress, we summarized the influence of drought, pathogen, and insect herbivory on stomatal regulation in some plant species. As one of the most detrimental abiotic stresses threating food security, drought is the most investigated stress for the cellular and molecular regulation of stomatal movement in plants. More than 90% of water in plants is lost through transpiration through stomatal pores (Pei et al., 1998); thus, stomatal regulation plays the vital role for plants to maintain water balance under drought conditions (Franks et al., 2007; Sperry et al., 2017). Therefore, we chose drought as the major representative abiotic stress and explore the crucial mutual mechanisms and crosstalk between abiotic and biotic stresses in the context of stomatal regulation in plants.

## Stomatal regulation and plant response to abiotic stresses

Stomatal response to individual abiotic stress such as drought (Chaves et al., 2009; Daszkowska-Golec and Szarejko, 2013; Martínez-Vilalta and Garcia-Forner, 2017; Gupta et al., 2020), light (Shimazaki et al., 2007; Matthews et al., 2020), heat (Sadok et al., 2021), and salinity (Aslam et al., 2011; Hedrich and Shabala, 2018) has been well studied and reviewed.

The mechanisms of drought-induced stomatal closure *via* the ABA signaling pathway have been summarized previously (Chen et al., 2017; Hauser et al., 2011; Hsu et al., 2021). Extensive studies on ABA signaling in the last 2 decades have been conducted around a chain of core signaling components. ABA receptors Pyrabactin Resistance (PYR) and Regulatory Component of ABA Receptor (RCAR) (Ma, 2009; Park et al., 2009) inhibit Protein Phosphatase 2Cs (PP2Cs) (Schweighofer et al., 2004) and promote the activation of Snf1-Related Protein Kinase 2 (SnRK2) kinases (Mustilli et al., 2002; Umezawa et al., 2009; Jalmi and Sinha, 2015), which target ion channels by inhibiting plasma membrane H^+^-ATPase and voltage-dependent K^+^ channels (Schroeder et al., 1987; Gao et al., 2017) as well as activating the S-type anion channels (SLAC) for stomatal closure (Hedrich and Geiger, 2017). The elevated ABA under drought conditions produces secondary messengers such as reactive oxygen species (ROS), nitric oxide (NO), and Ca^2+^. Elevated cytosolic Ca^2+^ activates Ca^2+^-dependent protein kinases (CDPK), phosphorylates PP2Cs, and also acts on slow anion channels such as SLAC1/SLAHs (Geiger et al., 2010). Interestingly, the production of ROS inhibits the activity of PP2Cs (Murata et al., 2001) and activates mitogen-activated protein kinases (MAPKs), which regulate the S-type anion channel for stomatal closure (Brock et al., 2010). ROS-activated MAPK signaling also functions in pathogen-triggered stomatal regulation, showing an excellent example for crosstalk between biotic and abiotic stresses (Jalmi and Sinha, 2015). Loss- or gain-of-function mutants of ABA signaling genes in *Arabidopsis*, rice, and other key crop species have laid a solid foundation for subsequent studies on stomatal response to other abiotic and biotic stresses.

## Stomata and plant innate immunity

Plants have evolved sophisticated strategies to perceive microbial infection and defense their attackers through an effective immune response. The role of stomata in plant innate immunity has been extensively reviewed (Melotto et al., 2008; Bharath et al., 2021). Stomata are usually the first line of defense against the pathogen, which restrict pathogen invasion by inducing stomatal closure or inhibiting stomatal opening (Melotto et al., 2006). Microbe/pathogen-associated molecular patterns (MAMPs/PAMPs) can induce stomatal closure within 1 h and the recognition of MAMPs by host cell transmembrane pattern recognition receptors (PRRs) [e.g. receptor kinases (RKs)] represents the initiation of evolutionarily conserved plant immune responses (Boller and Felix, 2009). The elevated salicylic acid (SA) level after pathogen invasion promotes the production of secondary messengers such as ROS, NO, and Ca^2+^ (Qi et al., 2018). These secondary messengers also induce the inactivation of the K^+^_in_ channel (Khokon et al., 2011) and the activation of SLAC1, leading to stomatal closure (Melotto et al., 2006; Segonzac et al., 2011). For instance, the immunity response of flagellin in *Arabidopsis* starts from the recognition of its highly conserved N-terminal epitope (flg22), which induces the heteromerization between a receptor kinase flagellin-sensitive 2 (FLS2) and Brassinosteroid Insensitive 1-associated Kinase 1 (BAK1) to activate innate plant immunity (Sun et al., 2013; Lozano-Duran et al., 2014). ABA is also required to induce stomatal closure during pathogen invasion (Miura and Tada, 2014) based on the results that stomatal closure is not found in plant response to flg22 either in the ABA-insensitive mutant *ost1* or in the ABA-deficient mutant *abi3-1* (Melotto et al., 2006). Therefore, we propose that a pathogen caused SA signaling to regulate at least the key downstream components (e.g. KAT1 and SLAC1) similar to those in the ABA signaling pathway under drought stress, but may also be linked to the upstream ABA reception modules such as OST1/SnRK2.6 protein kinase or ABI/PP2C protein phosphatase important for stomatal closure.

As counter-defense, virulence factors of pathogens have evolved to resist host plant stomatal defenses by blocking stomatal closure or inducing stomatal reopening (Melotto et al., 2006). For example, the plant pathogen *Pseudomonas syringae pv.* Tomato (*Pst*) DC3000 uses the virulence factor phytotoxin coronatine (COR) to reopen closed stomata (Melotto et al., 2006). It is proposed that the inhibition of COR on stomatal immune response is caused by promoting jasmonic acid (JA) production and SA deactivation since JA-SA antagonistic interactions have been one of the most characterized examples of phytohormone crosstalk (Aerts et al., 2021). COR also acts as a molecular mimic of JA-Ile and activates JA signaling by promoting a receptor complex formed by the F-box subunit COI1 of SCF-type Ubiquitin E3 Ligase (SCF^COI1^) and Jasmonate Zim Domain (JAZ) proteins. Then JAZ degradation (Sheard et al., 2010) activates transcriptional factors MYCs and NAC domain containing proteins (ANAC19, ANAC55, and ANAC72) for the potential inhibition of SA-mediated plant immunity against the bacteria, resulting in reopening of the stomata for pathogen invasion through stomatal pores (Zheng et al., 2012).

In addition to virulence factors, type-III-secretion-system effectors (T3SEs) of bacteria pathogens inhibit MAMP-triggered stomatal closure or promote stomatal opening (Zhou et al., 2011). The T3SE HopM1 of *P. syringae* disrupts the function of 14-3-3 protein GRF8/AtMIN10, leading to MAMP-triggered ROS burst (Lozano-Duran et al., 2014). Likewise, the HopF2 effector inhibits flg22-induced ROS by targeting RPM1-interacting protein (RIN4), which accelerates the activity of H^+^-ATPase (AHA) and ultimately inhibits stomatal closure (Hurley et al., 2014; Ray et al., 2019). The syringa-effector AvrB can also induce stomatal opening, which requires the JA signaling pathway to impair SA-triggered stomatal closure (Zhou et al., 2015). Moreover, HopX1 and HopZ1 effectors, reportedly from *P. syringae*, do not produce COR but also induce JAZ protein degradation, leading to stomatal opening (Bürger and Chory, 2019). Interestingly, 14-3-3 protein and H^+^-ATPase are two key components of stomatal opening, and the regulation of both proteins is important for ABA-induced stomatal closure and light-activated stomatal opening (Cotelle and Leonhardt, 2015; Wang Y. et al., 2018; Cai et al., 2021; Jiang et al., 2022). Therefore, the initiation of stomatal immunity upon pathogen invasion through the stomatal pore depends on some common secondary messengers, ion transporters, and regulatory proteins shared with the ABA signaling pathway for drought response in plants. Therefore, under pathogen invasion, the crosstalk between JA, SA, and ABA in stomatal guard cells is one of the key components for plant innate immunity. In summary, the multifunction and shared mechanisms of those molecular regulators in response to drought and pathogen stresses may have enabled resistance to single or combined stresses during the evolution of plants.

## Insect herbivory and stomatal response in plants: an overlooked plant-biotic interaction

Stomatal closure is a typical response of plants after herbivory damage (Pincebourde and Casas, 2006b; Schmidt et al., 2009; Nabity et al., 2013; Sun et al., 2015; Havko et al., 2020; Lin et al., 2021; Lin et al., 2022), but as to how plant stomata sense and conduct defense signals induced by insects, our understanding is still rudimentary. Chewing herbivores damage plant tissues to cause water loss through increased transpiration and sap-sucking herbivores decrease the water status of vascular tissues, which could both trigger stomatal closure (Sun et al., 2015). Therefore, herbivore-triggered stomatal closure could protect plants from water loss. For instance, *Operophtera brumata*, a common chewing herbivore in temperate forests, reduces stomatal conductance and photosynthesis in damaged leaves and neighbor undamaged leaves (Visakorpi et al., 2018), indicating that insect herbivory has major influences in modifying ecosystem carbon cycling. Similarly, fruit worm (*Helicoverpa zea*) significantly reduces the stomatal conductance of tomato (*Solanum lycopersicum*) and soybean (*Glycine max*) (Lin et al., 2021). There is a significant reduction of stomatal conductance after tobacco hornworm (*Manduca sexta*) feeding on wild-type tomato, but not in jasmonate-insensitive1 (*jai1-1*) tomato, indicating that JA-dependent wound response is related to plant stomatal movement when coping with insects (Havko et al., 2020). After sap-sucking insect *Bagrada hilaris* infestation, the photosynthesis and stomatal conductance of *Brassica oleracea* shows a continuous decline (Guarino et al., 2017). In summary, stomatal closure can possibly account for herbivore-induced reduction of photosynthesis that might implicate in the plant defense regulation against insect herbivore attack (Meza-Canales et al., 2017; Visakorpi et al., 2018).

Similar to PAMP-triggered plant immunity, multiple signaling components, including ROS, NO, Ca^2+^, and MAPKs, are activated after the perception of insect effectors. In response to insect herbivory, plants induce the defense processes regulated by receptors, phytohormones, secondary metabolites, and volatile compounds (Kerchev et al., 2012; Lin et al., 2022). Chewing insects cause the production of JA to induce Ca^2+^ signaling *via* CBL1-CIPK5 and GORK to mediate stomatal closure, which converges with the ABA signaling pathway (Adem et al., 2020). However, it is still unclear whether the stomatal closure is triggered by independent chewing insect-inflicted wounding or specific herbivore-associated molecular patterns (HAMPs) (Lin et al., 2021). SA has a more critical role in defensive response against piercing- and sucking-type insects than chewing ones (Bonaventure, 2012); upon insect attack, the SA-induced apoplastic burst of ROS is the first line of defense against subsequent attack. Among all types of ROS, H_2_O_2_ can disrupt the digestive system of insects, resulting in a shrinking insect herbivore population. Thus, it is one of the central components of defense response in plants against pests (War et al., 2011).

In addition to plant defense responses that triggered stomatal closure against insect attack, several components such as enzymes and pheromones that trigger stomatal closure have been identified in the oral secretions of herbivores. For example, the caterpillar *Helicoverpa zea* secretes salivary enzyme glucose oxidase (GOX) and causes stomatal closure in tomato and soybean leaves. GOX also suppresses the emission of herbivore-induced plant volatiles (HIPVs) during the feeding process, inhibiting airborne signals in plant defenses (Lin et al., 2021). The effector phospholipase C (PLC) in *caterpillar saliva* mediates the binding of inositol 1,4,5-triphosphate (IP_3_) and its receptor on the endoplasmic reticulum (ER), then triggers the rapid release of cytosolic Ca^2+^ (Turlings et al., 1990; Manaboon et al., 2009). Recent studies have shown the PLC-triggered signaling model is also related to the resistance of stomatal opening mediated by ABA (Cousson, 2008; Chamkhi et al., 2021). IP_3_ and Ca^2+^ are two important signaling components in stomatal closure (Gilroy et al., 1991; Ivanova et al., 2019), which could be the other important lines of evidence for the role of stomata in plant response to insect herbivory. These studies demonstrated that the virulence factors of pathogen prevent stomata from closing to facilitate pathogen entry into plants, whereas effectors of insect herbivores promote stomatal closure (Gilroy et al., 1991; Ivanova et al., 2019). The different mechanisms of multifactor stress interaction also indicate the potential competitive relationship between pathogen and pests for their infestation in plants.

## Stomatal closure: A double-edged sword for plants

**Figure 1:**
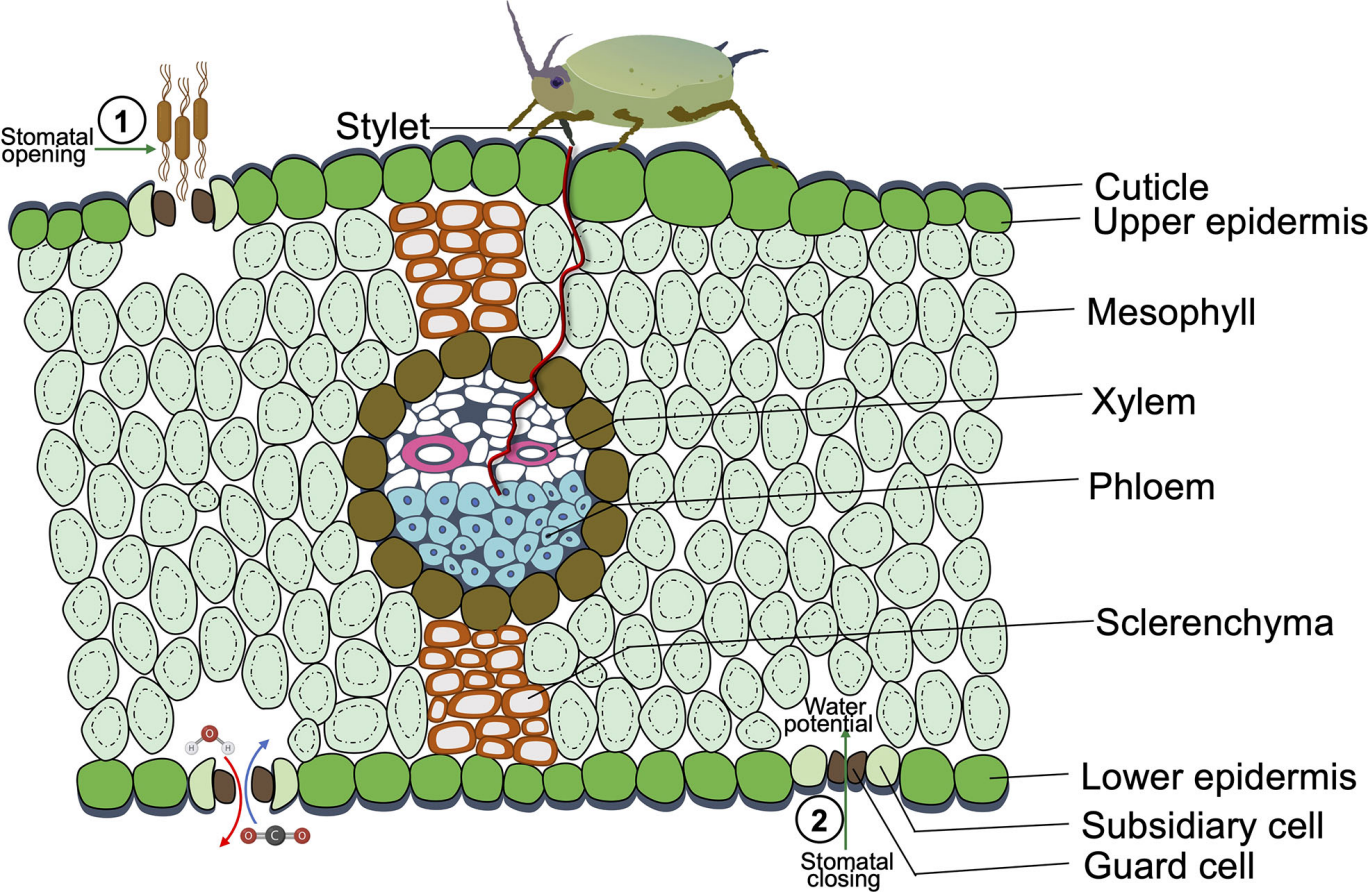
A Cross-sectional diagram of phloem sap-sucking insects feeding on a leaf of a monocot species. The opening of stomata favors pathogen invasion, as it eases the pathogen entry into plant tissues. While the sap-sucking insect infestation causes stomatal closure since sap-sucking insects feed on plant vascular tissues, mainly phloem, insects feed by penetrating their stylet in the epidermal tissue and eventually reach the sugary sap in the soft phloem tissue, which causes dehydration by water sucking and leads to the closure of guard cells. The herbivory of pest and pathogen infestation induces stomatal closure or opening, which potentially leads to the competitive relationship between pests and pathogens.

Manipulating stomatal closure is helpful for plants to respond to drought or pathogens, but it could have profound and robust benefits for herbivores (Lin et al., 2022). As the major gateways for photosynthetic CO_2_ assimilation and water transpiration, the key effects of insect herbivores on stomatal regulation are CO_2_ concentration, leaf temperature fluctuation, and water potential (**Figure 1**) (Block et al., 2017).

It has been revealed that the elevated level of atmospheric CO_2_ leads to an increase in herbivore and the population growth rate of aphids on plants, which is often accompanied by reduced stomatal aperture (Guo et al., 2013; Ryan et al., 2015; Li et al., 2019). It was suggested that the quick adaptation of insects to rising CO_2_ and warmer climates leads to more outbreaks of herbivorous insects under elevated global atmospheric CO_2_ that are devastating to plants and crops (Landsberg and Smith, 1992; Harvey et al., 2020). In addition, the gut pH of insects is more acidic in a warmer environment, and digests the food quickly, making the larval stages of many insect species more destructive to plants (Deutsch et al., 2018). Meanwhile, the stomatal aperture of most plant species is expected to decrease under elevated CO_2_, which further improves the performance of insects such as aphids, indicating that an elevated CO_2_ level will make the management of pests more arduous (Sun et al., 2015).

For leaf miners, there is an intimate contact with plant leaves coupled to leaf temperature, implying the link between stomata (major regulator of leaf temperature) and insect colonization (Pincebourde and Casas, 2006a). The physiology of ectothermic organisms, including insect herbivores, depends on microclimate temperature (Pincebourde and Casas, 2006b; Ma et al., 2018). The herbivore physically manipulates its proximate environment, especially plant tissues, and stomatal closure also regulates the leaf microclimate. Elevated temperature can trigger insect herbivore-induced JA, signaling block stomata opening and reduction of plant transpiration (Havko et al., 2020), which directly benefits insect herbivores by accelerating the growth (Barton, 2010), reducing the risk of predation (Urban, 2007; García-Robledo et al., 2016; García-Robledo and Baer, 2021).

Moreover, stomatal closure can maintain or even increase leaf water content to favor herbivores. For piercing-sucking insects, stomatal closure benefits them by fine-tuned leaf water potential. The aphid infestation triggered the stomatal closure of *Medicago truncatula*, causing decreased leaf transpiration and leaf water potential, which facilitates aphid infestation (Sun et al., 2015). More specifically, aphids feed on plant phloem saps, which are enriched with sugars and hold a four- to five-times greater osmotic pressure than aphid’s hemolymph (Douglas, 2006). Thus, aphids have to balance hemolymph osmolarity to avoid osmotic stress and self-dehydration during the feeding phase (Pompon et al., 2011). Therefore, it was speculated that stomatal closure of host plants can help aphids absorb more water from the xylem to neutralize phloem osmotic pressure (Guo et al., 2016) (**Figure 1**).

We speculate that compared with biotic stresses such as pathogen, insect feeding has a more adverse effect on plant physiological processes upon stomatal closure. Therefore, gaining comprehensive knowledge of stomatal mediated plant-herbivore interaction will be an important step towards the understanding of herbivores’ manipulation of plants and is beneficial for the development of integrated pest management. In the following section, we further discuss the effects of insect herbivory on plant stomata in the context of the existing molecular evidence on plants.

## Comparative transcriptome studies on plants under insect herbivore attack reveal a crosstalk between abiotic and biotic signaling pathways

**Figure 2:**
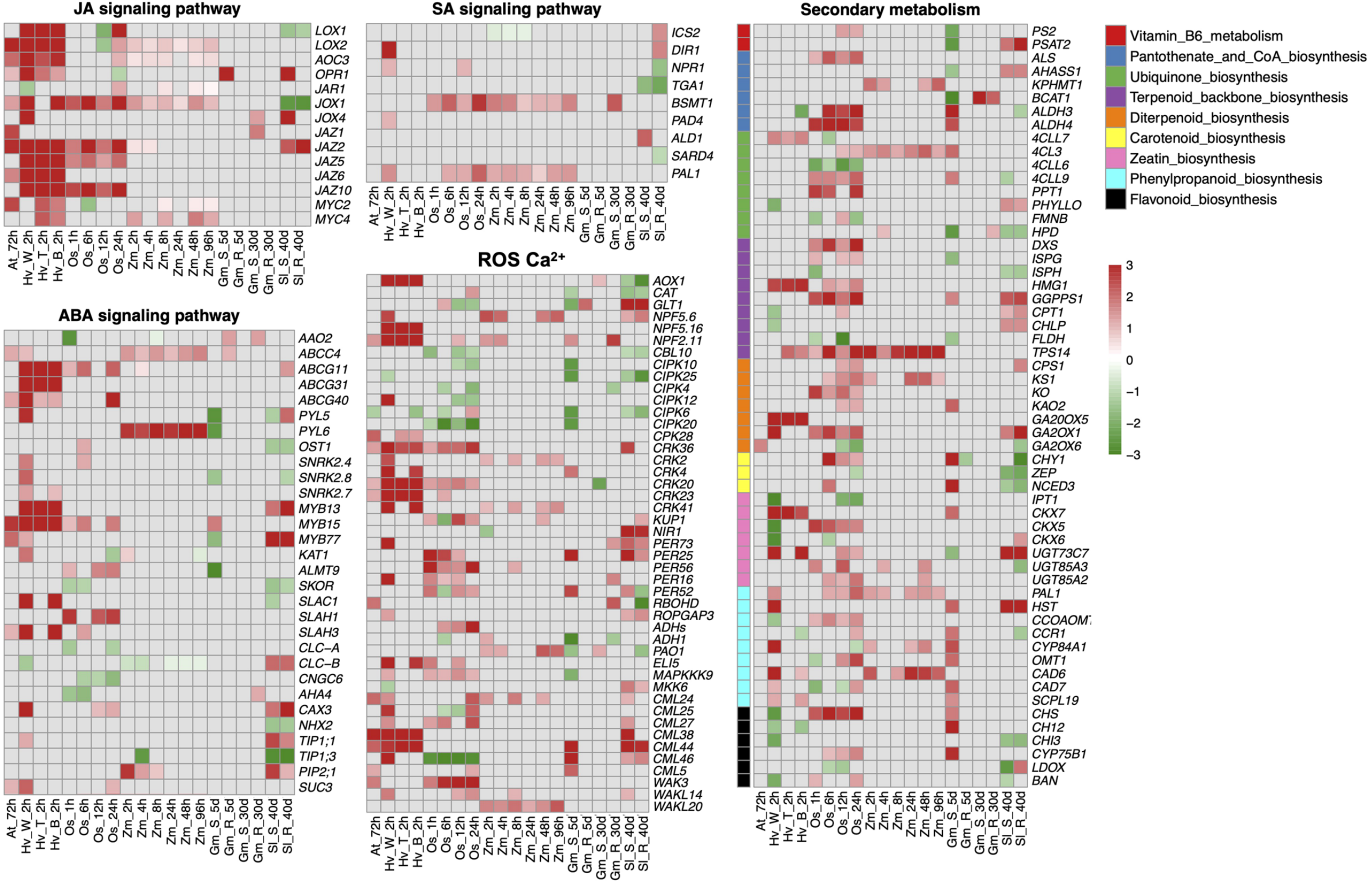
Integrative analysis of transcriptomes from different plant species fed by their main herbivores. The differential expressed genes (DEGs) with a threshold (fold change >1, FDR < 0.05) in JA, SA, ABA, ROS, and Ca^2+^ signaling pathways and plant secondary metabolisms (PSM) are shown in heatmaps. The color scale represents the fold change value from low (green) to high (red). The row name and column name of each heatmap indicate the gene name and experimental abbreviation, respectively. Abbreviations for experimental codes of plant and insect species can be found in **Table 1**. JA signaling pathway: *LOXs*, lipoxygenase; *AOCs*, allene oxide cyclase; *OPRs*, oxophytodienoic acid reductases; *JAR1*, jasmonate resistant 1; *JAZs*, jasmonate ZIM domain; *MYCs*, basic-helix-loop-helix transcription factor. ABA signaling pathway: *AAOs*, aba-aldehyde oxidase; *ABCGs*, Arabidopsis thaliana ATP-binding cassette G; *PYLs*, pyrabactin resistance-like; *SnRKs*, snf1-related protein protein kinase; *MYB*, transcription factor in abscisic acid signaling; *KATs*, guard cell inwardly rectifying K^+^ channel; *ALMTs*, aluminum-activated malate transporter; *SKORs*, outward-rectifier K^+^ channel; *SLAC/SLAHs*, S-type anion channels; *CLC-A/B*, chloride channel A/B; *CNGCs*, cyclic nucleotide-gated channels; *AHAs*, H^+^-ATPase; *CAXs*, cation/H^+^ exchanger; *NHXs*, sodium hydrogen exchanger; *TIPs*, tip growth defective; *PIPs*, plasma membrane intrinsic proteins; *SUCs*, Sucrose transport protein. SA signaling pathway: *ICSs*, isochorismate synthase; *DIRs*, drought-induced ring finger; *NPRs*, non-expressor of pathogenesis-related genes; *BSMT1*, BA/SA carboxyl methyltransferase 1; *PAD4*, peptidyl arginine deiminase 4; *SARD4*, SAR-deficient 4; *PAL1*, phenylalanine ammonia lyase. ROS, Ca^2+^: *AOXs*, alcohol oxidase; *CATs*, catalase; *NPFs*, nitrate peptide transporter family; *CIPKs*, CBL-interacting serine/threonine-protein kinase; *CRKs*, cysteine-rich receptor-like protein kinase; *KUPs*, potasium ion uptake permease; *NIRs*, nitrite reductase; *RBOHD*, respiratory burst oxidase homologous protein D; *ROPGAPs*, RHO GTPase activating proteins; *ADHs*, alcohol dehydrogenases; *PAOs*, polyamine oxidases; *MAPKs*, mitogen-activated protein kinase; *CMLs*, calmodulin-like proteins; *WAKs*, wall-associated receptor kinases. Secondary metabolism: *PS2*, phosphate starvation-induced gene 2; *PSAT2*, phosphoserine aminotransferase 2; *ALS*, acetolactate synthase; *AHASS1*, acetolactate synthase small subunit 1; *KPHMT1*, ketopantoate hydroxymethyltransferase 1; *BCAT1*, branched-chain amino acid transaminase 1; *ALDHs*, aldehyde dehydrogenase; *4CLs*, 4-coumarate-CoA ligase; *PPT1*, polyprenyltransferase 1; *PHYLLO*, 2-oxoglutarate decarboxylase/hydro-lyase/magnesium ion-binding protein; *FMNB*, fmn-binding protein; *HPD*, 4-hydroxyphenylpyruvate dioxygenase; *DXS*, 1-deoxy-d-xylulose 5-phosphate synthase; *ISPG*, (e)-4-hydroxy-3-methylbut-2-enyl-diphosphate synthase; *ISPH*, 4-hydroxy-3-methylbut-2-enyl diphosphate reductase; *HMG1*, hydroxy methylglutaryl CoA reductase 1; *GGPPS1*, geranylgeranyl diphosphate synthase 1; *CPT1*,cis-prenyltransferase 1; *CHLP*, geranylgeranyl reductase; *FLDH*, farnesol dehydrogenase; *TPS14*, terpene synthase 14; *CPS1*, copalyl diphosphate synthetase 1; *KS1*, kaurene synthase 1; *KO*, kaurene oxidase 1; *KAO2*, kaurenoic acid hydroxylase 2; *GA20OX5*, gibberellin 20-oxidase 5; *GA2OX1*, gibberellin 2-oxidase 1; *CHY1*, beta-hydroxyisobutyryl-coa hydrolase 1; *ZEP*, zeaxanthin epoxidase; *NCED3*, nine-cis-epoxycarotenoid dioxygenase 3; *IPT1*, isopentenyl transferase 1; *CKX5*, cytokinin oxidase 5; *UGTs*, UDP-glucosyl transferase; *HST*, homogentisate prenyltransferase; *CCOAOM*, caffeoyl coenzyme a ester o-methyltransferase; *CCR1*, cinnamoyl CoA reductase 1; *CYP84A1*, cytochrome p450 84A1; *OMT1*, o-methyltransferase 1; *CAD6*, cinnamyl alcohol dehydrogenase 6; *SCPL19*, serine carboxypeptidase-like 19; *CHS*, chalcone synthase; *CH12*, chlorina 12; *CYP75B1*, cytochrome p450 75B1; *LDOX*, leucoanthocyanidin dioxygenase; *BAN*, banyuls.

To further investigate the impacts of insect herbivores on plant stomatal regulation, we integrated RNA-sequencing datasets from different plant species including *Arabidopsis thaliana, Solanum lycopersicum, Oryza sativa, Glycine max, Hordeum vulgare*, and *Zea mays* infested by their main herbivores (**Table 1**). We combined these transcriptomes of different plant species using *Arabidopsis* homologs based on our previous publications (Chen et al., 2017; Zhao et al., 2019). The differentially expressed genes (DEGs) in JA, SA, and ABA signaling pathways, plant secondary metabolisms (PSM), and ROS and Ca^2+^ signaling pathways are shown in **Figure 2**.

**Table 1:** Integrated RNAseq datasets of different plant species attacked by their corresponding main pest.

Plant species	Herbivores	Treatment	Experiment code	Reference
*Arabidopsis thaliana*	*Myzus persicae*	aphid infested 72h	At_72h	(Annacondia et al., 2021)
*Solanum lycopersicum*	*Tuta absoluta*	Leaf miner fed 40d, susceptible genotype	Sl_S_40d	(D'Esposito et al., 2021)
*Solanum lycopersicum*	*Tuta absoluta*	leaf miner fed 40d, resistant genotype	Sl_R_40d	(D'Esposito et al., 2021)
*Oryza sativa*	*Cnaphalocrocis medinalis*	rice leaf roller fed 1h	Os_1h	(Wang et al., 2020)
*Oryza sativa*	*Cnaphalocrocis medinalis*	rice leaf roller fed 6h	Os _6h	(Wang et al., 2020)
*Oryza sativa*	*Cnaphalocrocis medinalis*	rice leaf roller fed 12h	Os _12h	(Wang et al., 2020)
*Oryza sativa*	*Cnaphalocrocis medinalis*	rice leaf roller fed 24h	Os _24h	(Wang et al., 2020)
*Glycine max*	*Aphis glycines*	aphid infested 5d, resistant genotype	Gm_R _5d	(Neupane et al., 2019)
*Glycine max*	*Aphis glycines*	aphid infested 30d, resistant genotype	Gm_R_30d	(Neupane et al., 2019)
*Glycine max*	*Aphis glycines*	aphid infested 5d, susceptible genotype	Gm_S _5d	(Neupane et al., 2019)
*Glycine max*	*Aphis glycines*	aphid infested 30d, susceptible genotype	Gm_S_30d	(Neupane et al., 2019)
*Hordeum vulgare*	*Tetranychus urticae*	TSSM-infested 2h	Hv_T_2h	(Bui et al., 2018)
*Hordeum vulgare*	*Oligonychus pratensis*	BGM-infested 2h	Hv_B_2h	(Bui et al., 2018)
*Hordeum vulgare*		leaf wounded 2h	Hv_W_2h	(Bui et al., 2018)
*Zea may*	*Rhopalosiphum maidis*	aphid infested 2h	Zm_2h	(Tzin et al., 2015)
*Zea may*	*Rhopalosiphum maidis*	aphid infested 4h	Zm_4h	(Tzin et al., 2015)
*Zea may*	*Rhopalosiphum maidis*	aphid infested 8h	Zm_8h	(Tzin et al., 2015)
*Zea may*	*Rhopalosiphum maidis*	aphid infested 24h	Zm_24h	(Tzin et al., 2015)
*Zea may*	*Rhopalosiphum maidis*	aphid infested 48h	Zm_48h	(Tzin et al., 2015)
*Zea may*	*Rhopalosiphum maidis*	aphid infested 96h	Zm_96h	(Tzin et al., 2015)

As expected, genes involved in JA biosynthesis, metabolism, and transport are widely differentially expressed in plant species after insect infestation, especially upregulating *MYC*, which promotes the biosynthesis of JA. The upregulation of *JAZs* indicates that the regulatory feedback loop involved MYC2 and JAZ proteins. Specifically, JAZ is degraded to activate MYCs after insect attack. In turn, activated MYC induces the up-regulation of JAZ and finally desensitizes plants (Howe and Yoshida, 2019). Likewise, lipoxygenase (LOX) involved in the first step in JA biosynthesis has been detected during responses to biotic and abiotic stresses in many plant species (Matsui, 2006). *LOX1*, expressed in guard cells, so-called green leaf volatiles, plays a key role in stomatal immunity *via* the SA-dependent and ABA-independent manner in response to both bacteria and the flg22 (Montillet and Hirt, 2013). The number of DEGs of the SA pathway is slightly less than those in the JA pathway where the most obvious ones are *Phenylalanine Ammonia Lyase* (*PAL1*) and *BA/SA carboxyl methyltransferase 1* (*BSMT1*) (**Figure 2**). PAL, the major enzyme in the phenylpropanoid pathway, also takes part in SA synthesis. However, the ABA level represses PAL activity in tomato (Audenaert et al., 2002) and soybean (Ward et al., 1989). SA can be converted into methyl-SA (MeSA) by BSMT1 (Attaran et al., 2009) and MeSA is an important herbivore-induced plant volatile to recruit natural enemies and finally defense-damaging pests (Rodriguez-Saona et al., 2011).

As the major phytohormone regulating stomata, DEGs were also found in the ABA signaling pathway in those plant transcriptome datasets after insect infestation treatments (**Figure 2**). The upregulation of ABA transporter *ABCG40* indicates there may be an enhanced ABA transport in guard cells after insect infestation (Kang et al., 2010). Both *MYC* and *MYB* proteins were upregulated, which function as transcriptional activators in ABA-inducible gene expression under drought stress in plants (Abe et al., 2003). However, plant species appear to respond differently in ABA signaling DEGs after insect infestation. For instance, herbivore-induced upregulation of *PYLs*, *SnRKs*, *SLAC/SLAH*, and *ALMTs* and downregulation of chloride channels *CLCa/CLCc* and *AHA* of *A. thaliana, O. sativa*, and *H. vulgare* are consistent with drought-induced stomatal closure (Cai et al., 2017; Eisenach and De Angeli, 2017). However, those DEGs were reversely expressed in a susceptible genotype of *G. max* after aphid infestation for 5 days and in *S. lycopersicum-*susceptible/resistant genotypes after leaf miner feeding for 40 days.

We also found DEGs involved in multiple signaling components, including ROS, NO, and Ca^2+^ signaling (**Figure 2**), which exist in guard cells to facilitate stomatal closure under biotic or abiotic stress (Ranty et al., 2016; Huang et al., 2019; Medeiros et al., 2020). Ca^2+^ sensors, including *CDPKs*, *calmodulin-like proteins (CMLs)* and *CBL (COBRA-like proteins)–CIPK (CBL-interacting serine/threonine-protein kinase)* complexes, are differentially expressed after herbivore infestation (**Figure 2**), among which *CBL–CIPK* complexes were downregulated in this study and reported negatively regulated ABA signaling during stomatal movement (Song et al., 2018). ROS scavenging is an important emerging mechanism for repairing damaged DNA or protein (Mittler, 2017), the *catalase (CATs)* and *peroxidase (PERs)* involved in ROS scavenging mechanisms were upregulated after herbivores infestation. The *cysteine-rich receptor-like protein kinases (CRKs)*, reported as a transcriptional regulator of pathogen-triggered immunity, ROS, and the SA signaling pathway, were highly upregulated after herbivore infestation (Acharya et al., 2007; Ederli et al., 2011). In addition, the central pillar of plant cells to sense and respond to the extracellular environment, *wall-associated receptor-like kinases (WAKs)*, were upregulated, indicating enhanced plant innate immunity to reconstruct ROS homeostasis (Delteil et al., 2016) and recognize effectors or DAMP through cell wall modification (Stephens et al., 2022).

Meanwhile, a wide range of DEGs are related to PSM, such as Vitamin B6 metabolism, ubiquinone biosynthesis, terpenoid biosynthesis, and phenylpropanoid biosynthesis. Plants have evolved several types of PSM to defend phytophagous herbivores such as alkaloids, terpenes, amines, glucosinolates, cyanogenic glucosides, quinones, phenolics, and polyacetylenes, through direct toxicity to pests and indirect protection by recruiting herbivorous natural enemies (Jamwal et al., 2018; Khare et al., 2020). Recent genetic and chemical studies have shown that PSM can induce the activity of JA and SA at a transcriptional level (Schweiger et al., 2014; Hettenhausen et al., 2015; Erb and Kliebenstein, 2020); similar results were observed in this study (**Figure 2**). The multifunction of PAL and LOX are excellent examples since the major enzyme in phenylpropanoid pathway, PAL, is also involved in SA synthesis (Smith et al., 2009; Klessig et al., 2016; Lefevere et al., 2020), and LOX plays a vital role in both JA biosynthesis and carotenoid biosynthesis (Balbi and Devoto, 2008; Smith et al., 2009; Shivaji et al., 2010; Liang et al., 2021). Terpenoid is the largest group of plant secondary metabolites; terpenes are active components in plant defense when plants are harmed by herbivores (Aharoni et al., 2005; Mumm et al., 2008; Wouters et al., 2016). Flavonoid natural compounds are insecticide synergists by destroying insect detoxification enzymes (Wang et al., 2016). In addition, the insecticidal potential of phenylpropanoids has been widely tested in different pest species (Sharma et al., 2006; Liu et al., 2013; Desmedt et al., 2021). Vitamin biosynthesis in plants is also a key target for novel pesticides because it is absent from animals (Smith et al., 2007); Vitamin B6 is involved in the biosynthesis of alkaloids (Drewke and Leistner, 2001). As one of the most promising components of pest management, alkaloids act as ingestion deterrent, growth inhibitor, and target neurotransmitter; affect neuronal signal transduction; and cause direct toxic effects on pests (Wink, 2012). In plants, pantothenate and CoA (Vitamin B5) are indispensable for lignin biosynthesis, which has been identified as a resistance factor against a number of insect species, including grasshoppers and caterpillars (Dowd et al., 2013). DEGs detected in these pathways are exactly mirrored in PSM regulation after insect feeding.

This transcriptomic analysis will offer great potential to identify key genes to bring novel insights into mechanisms underlying herbivore-induced stomatal regulation. Based on the integrated transcriptome datasets, it is worth noting that there are diverse plant responses to insects with different feeding modes (War et al., 2013). Here, we also found almost no DEGs of chosen pathways in *G. max* after aphid infestation for 30 days, but they were widely differentially expressed in *S. lycopersicum* after chewing herbivore (*Tuta absoluta*) feeding for a relative long-term 40 days. Therefore, whether insect feeding types or feeding time can affect plant defense response is worthy of further investigation.

## Are stomata important modulators of plant response to the stress combination?

Recent studies revealed that climate extremes will increase the complexity, frequency, and intensity of multiple stress combinations (Zandalinas et al., 2021), resulting in an increasing impact on plants from biotic stresses compounded by abiotic stress conditions (Havko et al., 2020). It implies the necessity to further study the stomatal regulation and plant plasticity under multiple stress combinations.

Plants develop complex mechanisms to cope with different abiotic and biotic stress with minimal cost (Mencuccini et al., 2019). It is difficult to predict how plants will deal with combined stress as a single stress condition is modified under combined stresses. The comparison of more than stresses combinations showed that each combined stress treatment results in a unique response in plants (Suzuki et al., 2014). The early perception of the stresses is crucial to activating an appropriate fine-tuning of the molecular pathways involved in stress response (Saijo and Loo, 2020). Plant stomata are important in shaping the overall responses of plants to the stress combination (Zandalinas et al., 2021). For example, a stomatal opening conduces to cool leaves through transpiration under heat stress, but closes to avoid water loss under drought. During a combination of heat stress and drought stress stomata of different plants remain closed, suggesting that drought-driven regulation of stomata overcomes heat stress-driven regulation (Zandalinas et al., 2021). Interestingly, an experiment was conducted recently; when high light and heat stress are treated to the same leaf of *Arabipdopsis* simultaneously, it does not induce changes in stomatal aperture; however, when these two stresses are treated to two different leaves, stomata display a rapid closing and then opening, indicating that heat stress-driven stomatal opening overcomes high light-driven stomatal closure during the stress combination (Zandalinas et al., 2020). Therefore, we proposed that stomata can function as important modulators of plant resistance to combined stress and play an important role in fine-tuning the crosstalk between different stress response pathways for plants to adapt to the changing environment.

As global warming is predicted to intensify more voracious arthropod pest populations, in the meantime may exacerbate crop losses. Stomatal closure benefits plants under pathogen invasion; it is worthwhile to investigate whether insect herbivore-induced stomatal closure will benefit plants or not. In a recent study, it was reported that herbivore-induced heat shock proteins motivate JA activity and inhibit stomatal opening resulting in leaf overheating and stunted plant growth at elevated temperatures (Havko et al., 2020). In addition, there is a potential competitive relationship between herbivore-induced stomatal closure and pathogen-induced stomatal reopening, since stomatal closure benefits insect feeding while the stomata pore is the first line for pathogen entry into leaves (**Figure 1**). For example, a salivary enzyme of insect herbivores, GOX, induces stomatal closure and may inhibit microbial invasion *via* producing H_2_O_2_ (Musser et al., 2005; Rojas et al., 2014).

**Figure 3:**
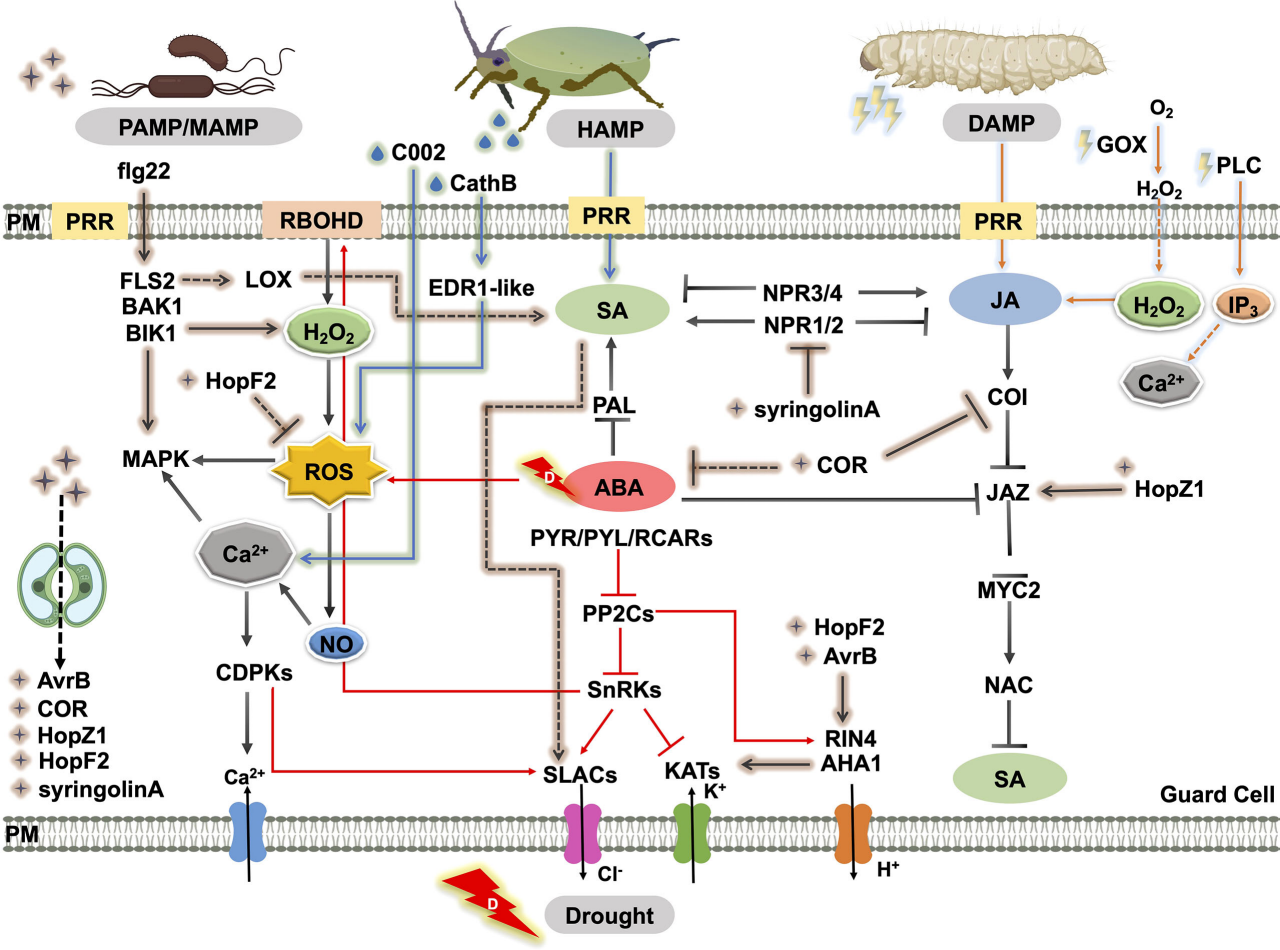
Schematic representation of the crosstalk of biotic and abiotic responses through the regulation of stomatal movement. MAMPs or PAMPs are perceived by receptors like PRRs. This recognition triggers plant immunity and elevates the SA level, which promotes the production of secondary messengers such as ROS, NO, and Ca^2+^. ROS accumulation leads to stomatal closure. As a counter defense, virulence factors or effectors produced by the pathogen such as COR, syringolinA, T3SEs, AvrB, HopX1, HopF2, and HopZ promote stomatal reopening by manipulating downstream ion channels or inhibitory effects on SA biosynthesis and SA signaling. Piercing or chewing insect attack triggers stomatal closure by mainly activating SA and JA signaling, respectively. Insect saliva enzymes and effectors such as GOX, PLC, CathB, and C002 also induce herbivory-induced stomatal closure *via* elevated ROS, Ca^2+^, and H_2_O_2_ levels. ABA affects insect-attack defense or pathogen-induced plant immunity by interacting with SA and JA. Straight and dashed arrows indicate direct and indirect interactions, respectively. Pointed and blunt arrows indicate activated and inhibited processes, respectively. Abbreviations, plant proteins: PLC, phospholipase c; RBOHD, respiratory burst oxidase homologou protein d; LOX, lipoxygenase; FLS2, flagellin-sensitive 2; BAK1, brassinosteroid insensitive 1-associated kinase 1; BIK1, Botrytis-Induced Kinase 1; MAPK, mitogen-activated protein kinase; CDPK, Ca^2+^-dependent protein kinases; PP2C, protein phosphatase 2C; SnRK, snf1-related protein protein kinase; SLAC, S-type anion channels; KAT, guard cell inwardly rectifying K^+^ channel; AHA, H^+^-ATPase; RIN4, rpm1 interacting protein 4; COI, coronatine insensitive; JAZ, jasmonate ZIM domain; MYC2, basic-helix-loop-helix transcription factor 2; NAC, NAC domain containing protein; PAL, phenylalanine ammonia lyase; PYR/PYL/RCAR, pyrabactin resistance/pyrabactin resistance-like/regulatory component of aba receptor; NPR, non-expressor of pathogenesis-related genes; ABCG, *Arabidopsis thaliana* ATP-binding cassette G; EDR1-like, enhanced disease resistance 1-like protein. Insect effectors: C002, effector detected in aphids; CathB, cathepsin b; GOX, glucose oxidase; PLC, thephospholipase C. Pathogen effectors: COR, coronatine; HopM1/HopF2, type-III-secretion-system effectors; XopR, effector of *Xanthomonas oryzae*; AvrB/HopZ1/HopX1, effector of *Syringa protolaciniata*.

In fact, the intricate signal regulatory networks that trigger stomatal movement under particular stress have conflicting effects under a combination of stresses, due to the clashing of hormonal signaling pathways and metabolic processes. In particular, they are embodied in the roles of SA, ABA, and JA in regulating plant defense responses and their interconnections (**Figure 3**), including the antagonistic crosstalk between SA and JA (Glazebrook, 2005) and SA and ABA (Cao et al., 2011), and the synergistic crosstalk between ABA and JA (Ju et al., 2019). Moreover, the trade-off under the particular stress of the individual hormone is still significant. For example, the SA signaling pathway mediates the release of plant volatiles to attract the natural enemies of insect pests, but SA-triggered stomatal closure can reduce the risk of finding insect predators (Peñaflor and Bento, 2013). The interactions discussed in the text are summarized in **Figure 3** to show the stomatal regulation in response to drought, pathogen, and insect herbivory.

## Conclusions and future perspectives

Stomata are at a crossroad of molecular interaction not limited to drought, pathogen, or insect herbivory, but also manipulated by underlying multiple stress combinations. This review highlights the influences and responses of stomatal opening or closing on pathogens and insect herbivores. Stomatal opening facilitates pathogen invasion and stomatal closure is stimulated by plant immunity response but in turn benefits insect infestation. Therefore, we proposed that insect herbivory has a more adverse effect on stomata-mediated physiological processes. To further investigate the impacts of herbivores on plant stomatal regulation, we integrated RNA-sequencing datasets from different plant species attacked by pests, which reveal some important interactions between abiotic and biotic signaling pathways embodied in phytohormone crosstalk and the multifunction of secondary messengers. Since the current understanding of stomata–stress interaction mechanisms are largely based on well-studied models such as the ABA signaling pathway, it is valuable to discuss the effect of different stimuli on stomata in the context of the existing molecular evidence.

Significant progress has been made in elucidating the molecular mechanisms of plants under single stress. However, how signal conflicts under multi-stress remains elusive. Stomata are at the center to perceive and respond to different environmental cues and play pivotal roles in orchestrating interactions between biotic and abiotic stresses. New insights of stomatal biology in the context of combined biotic and abiotic stress conditions are crucial for future plant biology research. Investigating stomatal response to multiple stresses represents an exciting frontier of plant science. Addressing these challenges will provide excellent perspectives for a broad and precise understanding of crosstalk between plant abiotic and biotic stress to shape plant–stress interactions at the molecular, physiological, and biochemical levels. The potential discoveries in these research areas will benefit our agriculture and environment in response to the current global climate changes.

## Author contributions

YW and Z-HC conceived and designed the research. PP and RL conducted the literature search. YW, Z-HC, and PP wrote the manuscript. All authors read and approved the final manuscript.

## Funding

This work is funded by the China Postdoctoral Science Foundation (2021M701355) and the Hubei Province Postdoctoral Innovation Positions Foundation.

## Acknowledgments

The computations in this paper were run on the bioinformatics computing platform of the National Key Laboratory of Crop Genetic Improvement, Huazhong Agricultural University.

## Conflict of interest

The authors declare that the research was conducted in the absence of any commercial or financial relationships that could be construed as a potential conflict of interest.

## Publisher’s note

All claims expressed in this article are solely those of the authors and do not necessarily represent those of their affiliated organizations, or those of the publisher, the editors and the reviewers. Any product that may be evaluated in this article, or claim that may be made by its manufacturer, is not guaranteed or endorsed by the publisher.
